# Structure of 2,2′-(5-*tert*-butyl-1,3-phenyl­ene)bis­(1-pentyl-1*H*-benzimidazol-3-ium) tetra­chlorido­mercurate(II)

**DOI:** 10.1107/S2056989017004303

**Published:** 2017-03-24

**Authors:** Varsha Rani, Harkesh B. Singh, Ray J. Butcher

**Affiliations:** a Department of Chemistry, Indian Institute of Technology Bombay, Powai, Mumbai 400 076, India; b Department of Chemistry, Howard University, 525 College Street NW, Washington, DC 20059, USA

**Keywords:** crystal structure, tetra­chlorido­mercurate(II) salt, (benzimidazol-2-yl)benzene ligands, hydrogen bonding

## Abstract

In the title salt, (C_34_H_44_N_4_)[HgCl_4_], the [C_34_H_44_N_4_]^2+^ cations and [HgCl_4_]^2−^ anions are linked by N—H⋯Cl hydrogen bonds. One of the two *n*-pentyl side chains was refined as disordered over two sets of sites, with occupancies of 0.733 (18) and 0.267 (18). The geometry around the Hg^II^ atom in the [HgCl_4_]^2−^ anion is distorted tetra­hedral, with bond angles ranging from 98.16 (3) to 120.68 (3)°. In the [HgCl_4_]^2−^ anion, there are two short Hg—Cl bonds [2.4120 (9) and 2.4171 (11) Å], one inter­mediate Hg—Cl bond [2.4716 (12) Å] and one long Hg—Cl bond [2.6579 (13) Å] for the Cl atom involved in a trifurcated hydrogen bond as an acceptor, including two N—H⋯Cl⋯H—N interactions as well as one C—H⋯Cl inter­action. There are several C—H⋯Cl inter­actions, with C⋯Cl distances ranging from 3.492 (3) to 3.796 (3) Å. These link the cations and anions into a zigzag chain along the *c*-axis direction. In addition, there are Cl⋯Cl halogen bonds, as well as π–π inter­actions, with centroid-to-centroid distances of 3.4765 (18) Å, which link one of the two benzimidazole moieties into dimeric units.

## Abstract 2

The structure of a 2,2′-[5-(*tert*-but­yl)-1,3-phenyl­ene)bis­(1-pentyl-1*H*-benzimidazol-3-ium) salt of the tetra­chlorido­mercurate(II) anion is reported in which there is an N—H⋯Cl⋯H—N trifurcated hydrogen bond.

## Chemical context   

During the past few years, metallated complexes of the ligand 1,3-bis­(1*H*-benzo[*d*]imidazol-2-yl)benzene have been well explored. This ligand is an ideal candidate for metalation due to the presence of two N atoms and one C atom, which bind tightly with metal atoms (Carina *et al.*, 1997 ▸; Obara *et al.*, 2006 ▸; Karlsson *et al.*, 2011 ▸; Yang *et al.*, 2012 ▸; Tam *et al.*, 2011 ▸; Gonzalez, 2014 ▸). As examples of the potential importance of this ligand, a highly phospho­rescent iridium complex with bis­(benzimidazol-2-yl)benzene ligand has been reported (Obara *et al.*, 2006 ▸) and helical and non helical copper(I) complexes with bis­(benzimidazol-2-yl)benzene have been described (Rüttimann *et al.*, 1992 ▸). A trimeric complex has been obtained through the self assembly of cyclo­metalated trinuclear palladium(II) complexes (Rüttimann *et al.*, 1993 ▸). Dinuclear zinc complexes containing a (benzimidazol-2-yl)benzene based ligand have shown anti­cancer activity (Xie *et al.*, 2014 ▸).

A literature survey of mercury halide complexes with benzimidazole derivatives has shown that they come in two main types: polymeric, bridging either through the halide (Zhang *et al.*, 2015 ▸; Li *et al.*, 2007 ▸; Shen *et al.*, 2005 ▸;) or through alternative N atoms from the benzimidazole moieties (Xiao *et al.*, 2009 ▸, 2011 ▸; Huang *et al.*, 2006 ▸; Li *et al.*, 2007 ▸, 2012*a* ▸,*b* ▸; Dey *et al.*, 2013 ▸; Du *et al.*, 2011 ▸; Chen *et al.*, 2013 ▸; Su *et al.*, 2003 ▸; Xu *et al.*, 2011 ▸); or as discrete mol­ecules (*i.e.* non-polymeric). Reports of structurally related complex have been published recently (Rani *et al.*, 2017*a* ▸,*b* ▸).

An attempt was made to synthesize the compound 2,2′-(5-(*tert*-but­yl)-2-(di­chloro­stiban­yl)-1,3-phenyl­ene)bis­(1-pentyl-1*H*-benzimidazole) (**2**) from (4-(*tert*-but­yl)-2,6-bis­(1-pentyl-1*H*-benzimidazol-2-yl)phen­yl)mercury(II) chloride; [C_34_H_41_N_4_HgCl] (**1**) using SbCl_3_ in dry 1,4-dioxane *via* transmetallation. Related reactions (Rani *et al.*, 2017*a* ▸,*b* ▸) had yielded complexes containing an Hg atom bound to the ligand through Hg—N bonds. However, it was observed that the crystallization of compound **2** in MeOH at room temperature led to the formation of a bis-benzimidazolium cation; [C_34_H_44_N_4_]^2+^[HgCl_4_]^2−^, **3**. The elaborate procedure for the synthesis of complex **1** will be published elsewhere.
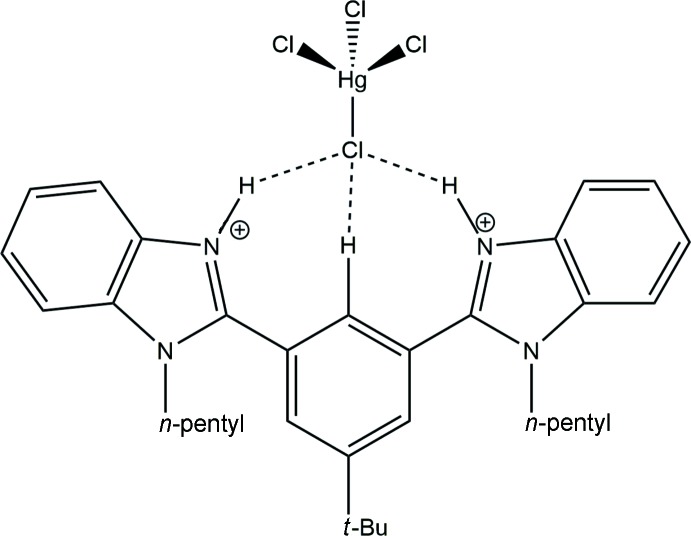


## Structural commentary   

**Figure 1:**
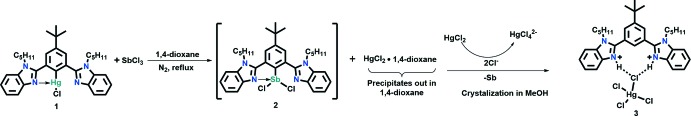
Reaction scheme showing the expected and actual products of the reaction.

**Figure 2:**
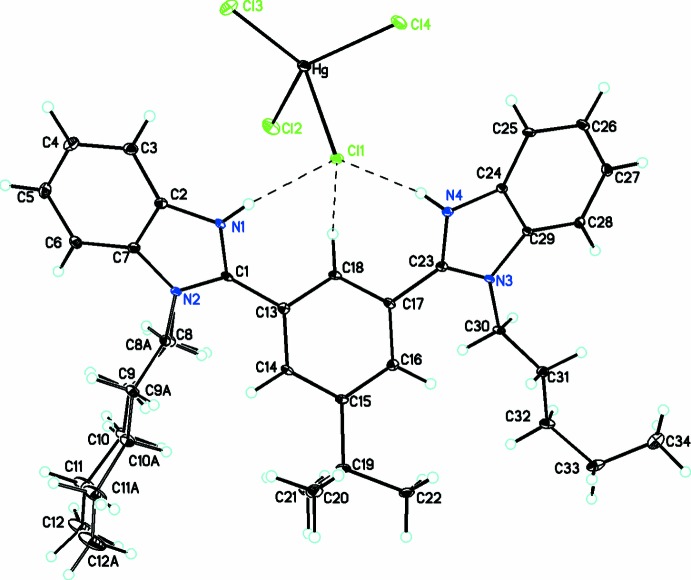
Diagram showing the atom labeling scheme, the trifurcated bond involving an N—H⋯Cl⋯H—N hydrogen bond, the C—H⋯Cl inter­actions and the disorder in one *n*-pentyl side chain. Atomic displacement parameters are at the 30% probability level.

The title compound, **3**, is a salt which contains [C_34_H_44_N_4_]^2+^ cations and [HgCl_4_]^2−^ anions linked by N—H⋯Cl hydrogen bonds. The reaction scheme leading to this product is shown in Fig. 1 ▸. The geometry around the mercury(II) atom in the [HgCl_4_]^2−^ anion is distorted tetra­hedral with bond angles ranging from 98.16 (3) to 120.68 (3)°. In the [HgCl_4_]^2−^ anion, there are two short Hg—Cl bonds [Hg—Cl4, 2.4120 (9) Å; Hg—Cl3, 2.4171 (11) Å], one inter­mediate Hg—Cl bond [Hg—Cl2, 2.4716 (12) Å] and one long Hg—Cl bond [Hg—Cl1, 2.6579 (13) Å] for the Cl atom involved in a trifurcated bond as an acceptor including two N—H⋯Cl⋯H—N interactions as well as one C—H⋯Cl inter­action (see Table 1 ▸), as shown in Fig. 2 ▸. Unlike a similar structure published recently containing a closely related ligand (Rani *et al.*, 2017*a* ▸), where the Hg atom is bonded to an N atom from the benzimidazole moiety, in this instance a salt has been obtained due to the different conditions of the reaction. The structure has been published of a salt containing the tetra­chlorido­mercurate(II) anion (Herbst *et al.*, 2013 ▸) and a closely related ligand with *n*-hexyl rather than *n*-pentyl side chains, which was the result of an attempted transmetallate reaction between Hg and Au.

In the ligand, the dihedral angles between the benzimidazole moieties and central phenyl ring are 40.60 (9) and 38.08 (10)°, while the angle between them is 36.04 (6)°. One of the pentyl substituents was refined as disordered over two sets of sites, with occupancies of 0.733 (18)/0.267 (18). The two pentyl side chains have adopted different conformations (for the disordered side-chain only values for the major conformation will be included) and this is illustrated by their torsion angles. For C8*A*–C12*A*, the angles involved, C1—N2—C8*A*—C9*A*, N2—C8*A*—C9*A*—C10*A*, C8*A*—C9*A*—C10*A*—C11*A*, and C9*A*—C10*A*—C11*A*—C12*A* and are 102.1 (16), −175.0 (15), 179.7 (15), and −178.1 (9)°, respectively, while for C30–C34 they are C23—N3—C30—C31, N3—C30—C31—C32, C30—C31—C32—C33, and C31—C32—C33—C34 [−105.7 (3), 175.7 (2), 173.0 (2) and −65.8 (3)°, respectively]. Thus the first side chain is in an all-*trans* conformation while the second side chain has adopted a conformation where it curls up at the end.

## Supra­molecular features   

**Figure 3:**
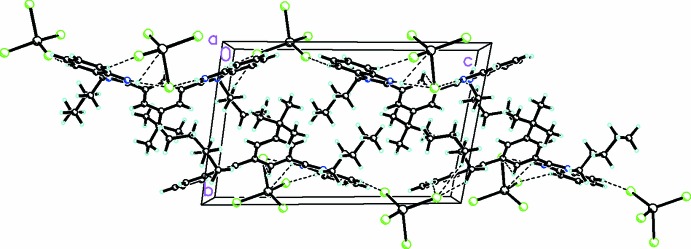
Diagram showing the C—H⋯Cl inter­actions, which link the cations and anions into a zigzag chain in the *c*-axis direction. The minor component of the pentyl disorder has been omitted for clarity. Atomic displacement parameters are at the 30% probability level.

**Figure 4:**
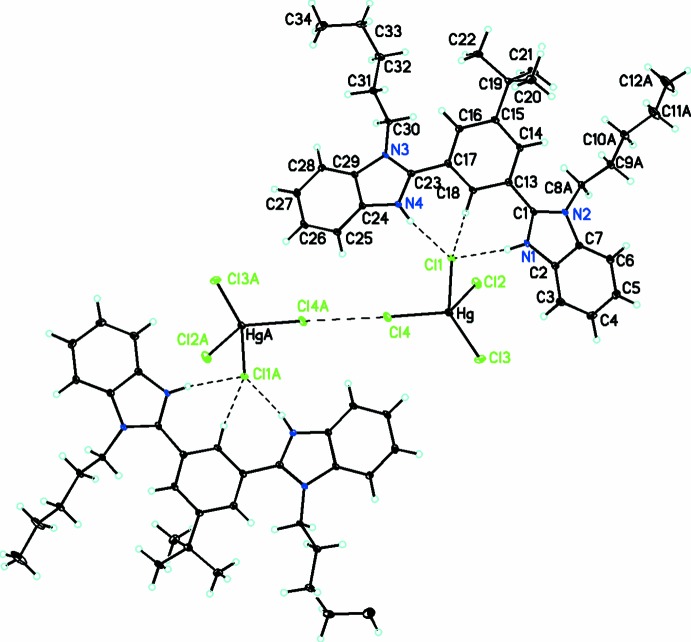
Diagram showing the Cl⋯Cl halogen bond.

In addition to the inter-ionic hydrogen bonds mentioned above, there are several C—H⋯Cl inter­actions with C⋯Cl distances ranging from 3.492 (3) to 3.796 (3) Å (see Table1). These link the cations and anions into a zigzag chain in the *c*-axis direction, as shown in Fig. 3 ▸. There are are Cl⋯Cl halogen bonds [Cl4⋯Cl42 − x, −y, 2 − z) = 3.434 (2) Å], as shown in Fig. 4 ▸. In addition, one of the two benzimidazole moieties forms dimeric units through π–π inter­actions (symmetry code 1 − *x*, −*y*, 2 − *z*) with centroid-to-centroid distances of 3.477 (2) Å.

## Database survey   

A survey of the Cambridge Structural database (CSD Version 5.37) for salts containing both the benzimidazole moiety as well as the tetra­chlorido­mercurate(II) anion gave eight hits, including a closely related ligand with *n*-hexyl rather than *n*-pentyl side chains (Herbst *et al.*, 2013 ▸).

## Synthesis and crystallization   

The reaction scheme is shown in Fig. 1 ▸. To a solution of **1** (0.2 g, 0.269 mmol) in dry 1,4-dioxane was added SbCl_3_ (0.061 g, 0.269 mmol) at room temperature. The reaction mixture was refluxed for 6 h under an inert atmosphere of N_2_ and filtered through Whatman filter paper. When the solvent was evaporated, a white-colored precipitate was obtained and purified by washing with hexane. The compound was dried under vacuum. Colourless block-shaped single crystals were obtained from MeOH at room temperature, yield 64% (0.120 g).

^1^H NMR (400 MHz, DMSO): δ 8.13 (*s*, 2H), 7.91 (*d*, *J* = 7.2 Hz, 2H), 7.82 (*d*, *J* = 7.2 Hz, 2H), 7.49–7.45 (*m*, 4H), 4.43 (*m*, 4H), 1.74 (*m*, 4H), 1.43 (*s*, 9H), 1.14 (*m*, 8H), 0.72 (*m*, 6H). ^13^C NMR (100 MHz, DMSO): 153.1, 151.4, 137.9, 134.7, 129.4, 128.5, 128.2, 124.7, 124.5, 117.8, 112.6, 45.1, 35.5, 31.3, 29.1, 28.5, 21.9, 14.1.

## Refinement   

Crystal data, data collection and structure refinement details are summarized in Table 2 ▸. One of the two *n*-pentyl side chains was refined as disordered over two sets of sites, with occupancies of 0.733 (18) and 0.267 (18) and both conformers were constrained to have similar metrical parameters using the SAME command in *SHELXL2016*. H atoms were positioned geometrically and refined as riding: N—H = 0.88 Å with *U*_iso_(H) = 1.2*U*_eq_(N); C—H = 0.95–0.98 Å with 1.2*U*_eq_(C) or 1.5*U*_eq_(C) for methyl H atoms.

## Supplementary Material

Crystal structure: contains datablock(s) I. DOI: 10.1107/S2056989017004303/zl2697sup1.cif

Structure factors: contains datablock(s) I. DOI: 10.1107/S2056989017004303/zl2697Isup2.hkl

CCDC reference: 1538746

Additional supporting information:  crystallographic information; 3D view; checkCIF report

## Figures and Tables

**Table 1:** Hydrogen-bond geometry (Å, °)

*D*—H⋯*A*	*D*—H	H⋯*A*	*D*⋯*A*	*D*—H⋯*A*
N1—H1*A*⋯Cl1	0.88	2.30	3.171 (2)	171
N4—H4*B*⋯Cl1	0.88	2.35	3.224 (2)	170
C3—H3*A*⋯Cl3	0.95	2.90	3.803 (3)	160
C6—H6*A*⋯Cl2^i^	0.95	2.56	3.492 (3)	169
C18—H18*A*⋯Cl1	0.95	2.85	3.331 (4)	113
C25—H25*A*⋯Cl4^ii^	0.95	2.96	3.664 (3)	132
C28—H28*A*⋯Cl4^iii^	0.95	2.91	3.796 (3)	156
C30—H30*A*⋯Cl4^iii^	0.99	2.77	3.627 (3)	145

Symmetry codes: (i) 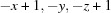; (ii) 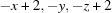; (iii) 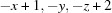.

**Table 2:** Experimental details

Crystal data	
Chemical formula	(C_34_H_44_N_4_)[HgCl_4_]
*M*_r_	851.12
Crystal system, space group	Triclinic, *P* 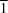
Temperature (K)	100
*a*, *b*, *c* (Å)	9.806 (5), 11.264 (5), 17.274 (5)
α, β, γ (°)	96.727 (5), 95.859 (5), 108.575 (5)
*V* (Å^3^)	1776.4 (13)
*Z*	2
Radiation type	Cu *K*α
μ (mm^−1^)	10.76
Crystal size (mm)	0.20 × 0.11 × 0.09

Data collection	
Diffractometer	Bruker Quest CCD
Absorption correction	Multi-scan (*SADABS*; Sheldrick, 1996 ▸)
*T*_min_, *T*_max_	0.497, 0.753
No. of measured, independent and observed [*I* > 2σ(*I*)] reflections	6193, 6193, 6138
*R*_int_	0.038
(sin θ/λ)_max_ (Å^−1^)	0.595

Refinement	
*R*[*F* ^2^ > 2σ(*F* ^2^)], *wR*(*F* ^2^), *S*	0.020, 0.048, 1.10
No. of reflections	6193
No. of parameters	410
No. of restraints	67
H-atom treatment	H-atom parameters constrained
Δρ_max_, Δρ_min_ (e Å^−3^)	1.36, −0.76

Computer programs: *APEX2* (Bruker, 2005 ▸), *SAINT* (Bruker, 2002 ▸), *SIR92* (Altomare *et al.*, 1993 ▸), *SHELXL2016* (Sheldrick, 2015 ▸) and *SHELXTL* (Sheldrick, 2008 ▸).

## supplementary crystallographic information

### Crystal data

**Table:**

(C_34_H_44_N_4_)[HgCl_4_]	*Z* = 2
*M**_r_* = 851.12	*F*(000) = 848
Triclinic, *P*1	*D*_x_ = 1.591 Mg m^−^^3^
*a* = 9.806 (5) Å	Cu *K*α radiation, λ = 1.54178 Å
*b* = 11.264 (5) Å	Cell parameters from 9629 reflections
*c* = 17.274 (5) Å	θ = 2.6–61.2°
α = 96.727 (5)°	µ = 10.76 mm^−^^1^
β = 95.859 (5)°	*T* = 100 K
γ = 108.575 (5)°	Block, colourless
*V* = 1776.4 (13) Å^3^	0.20 × 0.11 × 0.09 mm

### Data collection

**Table:**

Bruker Quest CCD diffractometer	6138 reflections with *I* > 2σ(*I*)
ω scans	*R*_int_ = 0.038
Absorption correction: multi-scan (SADABS; Sheldrick, 1996)	θ_max_ = 66.6°, θ_min_ = 2.6°
*T*_min_ = 0.497, *T*_max_ = 0.753	*h* = −11→11
6193 measured reflections	*k* = −13→13
6193 independent reflections	*l* = −20→20

### Refinement

**Table:**

Refinement on *F*^2^	67 restraints
Least-squares matrix: full	Hydrogen site location: mixed
*R*[*F*^2^ > 2σ(*F*^2^)] = 0.020	H-atom parameters constrained
*wR*(*F*^2^) = 0.048	*w* = 1/[σ^2^(*F*_o_^2^) + (0.0123*P*)^2^ + 4.1059*P*] where *P* = (*F*_o_^2^ + 2*F*_c_^2^)/3
*S* = 1.10	(Δ/σ)_max_ = 0.002
6193 reflections	Δρ_max_ = 1.36 e Å^−^^3^
410 parameters	Δρ_min_ = −0.76 e Å^−^^3^

### Special details

**Table:**

Geometry. All esds (except the esd in the dihedral angle between two l.s. planes) are estimated using the full covariance matrix. The cell esds are taken into account individually in the estimation of esds in distances, angles and torsion angles; correlations between esds in cell parameters are only used when they are defined by crystal symmetry. An approximate (isotropic) treatment of cell esds is used for estimating esds involving l.s. planes.

### Fractional atomic coordinates and isotropic or equivalent isotropic displacement parameters (Å^2^)

**Table:**

	*x*	*y*	*z*	*U*_iso_*/*U*_eq_	Occ. (<1)
Hg	0.92160 (2)	0.04332 (2)	0.77777 (2)	0.01320 (4)	
Cl1	0.90325 (6)	0.27221 (6)	0.81804 (3)	0.01173 (12)	
Cl2	0.67643 (7)	−0.05962 (7)	0.70059 (4)	0.02181 (15)	
Cl3	1.13497 (7)	0.09387 (7)	0.71258 (4)	0.02149 (14)	
Cl4	0.92562 (7)	−0.03357 (7)	0.90227 (4)	0.02064 (14)	
N1	0.7288 (2)	0.2251 (2)	0.64598 (12)	0.0103 (4)	
H1A	0.786478	0.240862	0.691195	0.012*	
N2	0.5412 (2)	0.2067 (2)	0.55973 (12)	0.0093 (4)	
N3	0.4506 (2)	0.2061 (2)	0.96891 (12)	0.0083 (4)	
N4	0.6596 (2)	0.2112 (2)	0.93340 (12)	0.0093 (4)	
H4B	0.731909	0.222420	0.905661	0.011*	
C1	0.5974 (3)	0.2376 (2)	0.63675 (15)	0.0093 (5)	
C2	0.7605 (3)	0.1834 (2)	0.57342 (15)	0.0107 (5)	
C3	0.8791 (3)	0.1515 (3)	0.55187 (16)	0.0145 (5)	
H3A	0.959906	0.157791	0.589417	0.017*	
C4	0.8719 (3)	0.1104 (3)	0.47260 (17)	0.0175 (6)	
H4A	0.950066	0.087255	0.455158	0.021*	
C5	0.7527 (3)	0.1016 (3)	0.41676 (17)	0.0182 (6)	
H5A	0.753552	0.074077	0.362738	0.022*	
C6	0.6347 (3)	0.1318 (3)	0.43830 (16)	0.0160 (6)	
H6A	0.553716	0.125044	0.400717	0.019*	
C7	0.6410 (3)	0.1728 (2)	0.51834 (16)	0.0112 (5)	
C8	0.388 (7)	0.196 (6)	0.529 (4)	0.011 (2)	0.267 (18)
H8A	0.324342	0.184164	0.568393	0.013*	0.267 (18)
H8B	0.347222	0.123574	0.487932	0.013*	0.267 (18)
C9	0.383 (3)	0.295 (4)	0.477 (3)	0.0131 (15)	0.267 (18)
H9A	0.441717	0.290281	0.434405	0.016*	0.267 (18)
H9B	0.425475	0.380907	0.509106	0.016*	0.267 (18)
C10	0.226 (3)	0.274 (2)	0.4416 (15)	0.0146 (14)	0.267 (18)
H10A	0.185328	0.190262	0.407582	0.018*	0.267 (18)
H10B	0.166182	0.274397	0.484450	0.018*	0.267 (18)
C11	0.218 (2)	0.377 (2)	0.3929 (14)	0.0331 (16)	0.267 (18)
H11A	0.261683	0.461609	0.426420	0.040*	0.267 (18)
H11B	0.274690	0.375184	0.348875	0.040*	0.267 (18)
C12	0.061 (2)	0.359 (2)	0.3600 (16)	0.045 (2)	0.267 (18)
H12A	0.059390	0.417668	0.322680	0.067*	0.267 (18)
H12B	0.008610	0.375105	0.403260	0.067*	0.267 (18)
H12C	0.013309	0.271338	0.332829	0.067*	0.267 (18)
C8A	0.396 (2)	0.198 (2)	0.5222 (15)	0.011 (2)	0.733 (18)
H8AA	0.336219	0.197140	0.562771	0.013*	0.733 (18)
H8AB	0.355259	0.119230	0.486591	0.013*	0.733 (18)
C9A	0.3993 (11)	0.3134 (11)	0.4833 (9)	0.0131 (15)	0.733 (18)
H9AA	0.457776	0.317383	0.439655	0.016*	0.733 (18)
H9AB	0.445820	0.391532	0.522333	0.016*	0.733 (18)
C10A	0.2453 (8)	0.3062 (7)	0.4513 (5)	0.0146 (14)	0.733 (18)
H10C	0.199418	0.227719	0.412483	0.018*	0.733 (18)
H10D	0.187130	0.301002	0.495179	0.018*	0.733 (18)
C11A	0.2417 (7)	0.4193 (8)	0.4123 (5)	0.0331 (16)	0.733 (18)
H11C	0.290625	0.498002	0.450600	0.040*	0.733 (18)
H11D	0.297192	0.422840	0.367341	0.040*	0.733 (18)
C12A	0.0887 (7)	0.4149 (10)	0.3828 (6)	0.045 (2)	0.733 (18)
H12D	0.093526	0.487533	0.355660	0.067*	0.733 (18)
H12E	0.035429	0.418294	0.427505	0.067*	0.733 (18)
H12F	0.038428	0.335987	0.346036	0.067*	0.733 (18)
C13	0.5319 (3)	0.2776 (2)	0.70281 (15)	0.0096 (5)	
C14	0.4612 (3)	0.3667 (2)	0.69900 (15)	0.0106 (5)	
H14A	0.448566	0.397654	0.650880	0.013*	
C15	0.4086 (3)	0.4111 (2)	0.76422 (15)	0.0099 (5)	
C16	0.4336 (3)	0.3674 (2)	0.83453 (15)	0.0100 (5)	
H16A	0.402656	0.399307	0.880457	0.012*	
C17	0.5035 (3)	0.2771 (2)	0.83886 (15)	0.0090 (5)	
C18	0.5510 (3)	0.2304 (2)	0.77264 (15)	0.0092 (5)	
H18A	0.595734	0.167208	0.774895	0.011*	
C19	0.3288 (3)	0.5074 (2)	0.75636 (16)	0.0139 (5)	
C20	0.4329 (4)	0.6272 (3)	0.73242 (19)	0.0228 (7)	
H20A	0.382906	0.689130	0.726752	0.034*	
H20B	0.463592	0.604517	0.682141	0.034*	
H20C	0.518497	0.664220	0.773123	0.034*	
C21	0.1958 (3)	0.4478 (3)	0.69226 (18)	0.0243 (7)	
H21A	0.131693	0.369865	0.706788	0.036*	
H21B	0.227761	0.427582	0.641934	0.036*	
H21C	0.142904	0.507698	0.687084	0.036*	
C22	0.2771 (3)	0.5445 (3)	0.83319 (17)	0.0162 (6)	
H22A	0.208836	0.468930	0.847978	0.024*	
H22B	0.228501	0.606900	0.825887	0.024*	
H22C	0.360949	0.581275	0.874997	0.024*	
C23	0.5353 (3)	0.2327 (2)	0.91271 (15)	0.0088 (5)	
C24	0.6564 (3)	0.1682 (2)	1.00576 (15)	0.0091 (5)	
C25	0.7573 (3)	0.1319 (2)	1.05176 (15)	0.0112 (5)	
H25A	0.847917	0.133746	1.036094	0.013*	
C26	0.7169 (3)	0.0929 (2)	1.12160 (16)	0.0127 (5)	
H26A	0.782433	0.068395	1.155286	0.015*	
C27	0.5816 (3)	0.0886 (2)	1.14430 (15)	0.0126 (5)	
H27A	0.558408	0.060958	1.192707	0.015*	
C28	0.4818 (3)	0.1234 (2)	1.09797 (15)	0.0104 (5)	
H28A	0.390052	0.119514	1.112863	0.013*	
C29	0.5231 (3)	0.1643 (2)	1.02846 (15)	0.0086 (5)	
C30	0.3016 (3)	0.2106 (2)	0.96844 (15)	0.0097 (5)	
H30A	0.239454	0.132290	0.984765	0.012*	
H30B	0.260795	0.213677	0.914165	0.012*	
C31	0.2987 (3)	0.3254 (2)	1.02351 (15)	0.0116 (5)	
H31A	0.366003	0.403763	1.009825	0.014*	
H31B	0.331720	0.319240	1.078541	0.014*	
C32	0.1447 (3)	0.3319 (3)	1.01653 (17)	0.0153 (6)	
H32A	0.117316	0.347650	0.962901	0.018*	
H32B	0.076545	0.248586	1.023004	0.018*	
C33	0.1271 (3)	0.4344 (3)	1.07662 (18)	0.0209 (6)	
H33A	0.198659	0.517088	1.071842	0.025*	
H33B	0.028751	0.439720	1.063744	0.025*	
C34	0.1476 (4)	0.4122 (3)	1.16171 (19)	0.0294 (7)	
H34A	0.123242	0.475748	1.196056	0.044*	
H34B	0.249058	0.419258	1.177643	0.044*	
H34C	0.083626	0.327163	1.166161	0.044*	

### Atomic displacement parameters (Å^2^)

**Table:**

	*U*^11^	*U*^22^	*U*^33^	*U*^12^	*U*^13^	*U*^23^
Hg	0.01221 (6)	0.01533 (7)	0.01315 (6)	0.00693 (5)	−0.00075 (4)	0.00220 (4)
Cl1	0.0100 (3)	0.0128 (3)	0.0126 (3)	0.0058 (2)	−0.0012 (2)	−0.0006 (2)
Cl2	0.0157 (3)	0.0210 (3)	0.0217 (3)	0.0007 (3)	−0.0083 (3)	0.0010 (3)
Cl3	0.0168 (3)	0.0241 (4)	0.0258 (4)	0.0097 (3)	0.0075 (3)	0.0008 (3)
Cl4	0.0176 (3)	0.0293 (4)	0.0191 (3)	0.0108 (3)	0.0016 (3)	0.0121 (3)
N1	0.0104 (10)	0.0146 (11)	0.0073 (10)	0.0067 (9)	−0.0015 (8)	0.0027 (8)
N2	0.0096 (10)	0.0109 (11)	0.0087 (10)	0.0056 (9)	−0.0003 (8)	0.0019 (8)
N3	0.0082 (10)	0.0088 (10)	0.0088 (10)	0.0046 (8)	−0.0010 (8)	0.0016 (8)
N4	0.0084 (10)	0.0107 (11)	0.0102 (11)	0.0046 (9)	0.0018 (8)	0.0024 (8)
C1	0.0114 (12)	0.0061 (12)	0.0102 (12)	0.0030 (10)	−0.0008 (10)	0.0033 (9)
C2	0.0105 (12)	0.0097 (12)	0.0120 (13)	0.0042 (10)	0.0005 (10)	0.0016 (10)
C3	0.0109 (13)	0.0167 (14)	0.0173 (14)	0.0064 (11)	0.0010 (11)	0.0040 (11)
C4	0.0144 (13)	0.0195 (15)	0.0212 (15)	0.0082 (12)	0.0064 (11)	0.0026 (12)
C5	0.0231 (15)	0.0205 (15)	0.0123 (14)	0.0094 (12)	0.0044 (11)	0.0002 (11)
C6	0.0189 (14)	0.0208 (15)	0.0102 (13)	0.0105 (12)	−0.0012 (11)	0.0020 (11)
C7	0.0124 (13)	0.0101 (12)	0.0124 (13)	0.0052 (10)	0.0017 (10)	0.0022 (10)
C8	0.007 (3)	0.0168 (16)	0.008 (5)	0.0046 (19)	−0.001 (3)	0.002 (2)
C9	0.009 (2)	0.016 (4)	0.014 (3)	0.003 (2)	−0.002 (2)	0.005 (3)
C10	0.008 (2)	0.017 (4)	0.017 (3)	0.003 (3)	−0.0042 (17)	0.003 (3)
C11	0.015 (2)	0.037 (4)	0.050 (4)	0.009 (3)	−0.003 (2)	0.026 (3)
C12	0.021 (3)	0.046 (5)	0.070 (5)	0.010 (3)	−0.007 (3)	0.036 (4)
C8A	0.007 (3)	0.0168 (16)	0.008 (5)	0.0046 (19)	−0.001 (3)	0.002 (2)
C9A	0.009 (2)	0.016 (4)	0.014 (3)	0.003 (2)	−0.002 (2)	0.005 (3)
C10A	0.008 (2)	0.017 (4)	0.017 (3)	0.003 (3)	−0.0042 (17)	0.003 (3)
C11A	0.015 (2)	0.037 (4)	0.050 (4)	0.009 (3)	−0.003 (2)	0.026 (3)
C12A	0.021 (3)	0.046 (5)	0.070 (5)	0.010 (3)	−0.007 (3)	0.036 (4)
C13	0.0081 (12)	0.0084 (12)	0.0105 (12)	0.0014 (10)	−0.0007 (10)	0.0004 (10)
C14	0.0112 (12)	0.0098 (12)	0.0113 (13)	0.0039 (10)	−0.0007 (10)	0.0043 (10)
C15	0.0094 (12)	0.0067 (12)	0.0135 (13)	0.0028 (10)	0.0006 (10)	0.0024 (10)
C16	0.0102 (12)	0.0077 (12)	0.0111 (13)	0.0022 (10)	0.0010 (10)	0.0003 (10)
C17	0.0075 (12)	0.0072 (12)	0.0101 (12)	0.0004 (10)	−0.0019 (9)	0.0018 (10)
C18	0.0068 (12)	0.0076 (12)	0.0128 (13)	0.0025 (10)	−0.0012 (10)	0.0023 (10)
C19	0.0182 (14)	0.0101 (13)	0.0174 (14)	0.0095 (11)	0.0030 (11)	0.0042 (10)
C20	0.0355 (18)	0.0146 (14)	0.0269 (16)	0.0148 (13)	0.0148 (14)	0.0104 (12)
C21	0.0270 (16)	0.0317 (17)	0.0223 (16)	0.0234 (14)	−0.0040 (13)	0.0036 (13)
C22	0.0211 (14)	0.0128 (13)	0.0211 (15)	0.0118 (12)	0.0075 (12)	0.0060 (11)
C23	0.0097 (12)	0.0047 (12)	0.0106 (12)	0.0017 (10)	−0.0005 (10)	−0.0001 (9)
C24	0.0122 (12)	0.0054 (12)	0.0090 (12)	0.0037 (10)	−0.0013 (10)	−0.0007 (9)
C25	0.0107 (12)	0.0100 (13)	0.0136 (13)	0.0060 (10)	−0.0012 (10)	0.0004 (10)
C26	0.0156 (13)	0.0093 (13)	0.0132 (13)	0.0063 (11)	−0.0044 (10)	0.0009 (10)
C27	0.0185 (14)	0.0093 (12)	0.0099 (13)	0.0047 (11)	0.0010 (10)	0.0021 (10)
C28	0.0132 (13)	0.0079 (12)	0.0099 (12)	0.0042 (10)	0.0011 (10)	−0.0006 (10)
C29	0.0100 (12)	0.0057 (12)	0.0091 (12)	0.0033 (10)	−0.0030 (9)	−0.0011 (9)
C30	0.0049 (12)	0.0120 (13)	0.0128 (13)	0.0037 (10)	−0.0008 (9)	0.0031 (10)
C31	0.0096 (12)	0.0130 (13)	0.0124 (13)	0.0050 (10)	−0.0003 (10)	0.0014 (10)
C32	0.0121 (13)	0.0146 (14)	0.0204 (14)	0.0070 (11)	−0.0007 (11)	0.0027 (11)
C33	0.0188 (15)	0.0177 (15)	0.0300 (17)	0.0121 (12)	0.0044 (12)	0.0011 (12)
C34	0.0379 (19)	0.0280 (17)	0.0275 (17)	0.0191 (15)	0.0084 (14)	−0.0012 (14)

### Geometric parameters (Å, º)

**Table:**

Hg—Cl4	2.4120 (9)	C11A—H11C	0.9900
Hg—Cl3	2.4171 (11)	C11A—H11D	0.9900
Hg—Cl2	2.4716 (12)	C12A—H12D	0.9800
Hg—Cl1	2.6579 (13)	C12A—H12E	0.9800
Cl4—Cl4^i^	3.4343 (16)	C12A—H12F	0.9800
N1—C1	1.336 (3)	C13—C14	1.393 (4)
N1—C2	1.386 (3)	C13—C18	1.393 (4)
N1—H1A	0.8800	C14—C15	1.392 (4)
N2—C1	1.347 (3)	C14—H14A	0.9500
N2—C7	1.390 (3)	C15—C16	1.391 (4)
N2—C8A	1.468 (17)	C15—C19	1.536 (3)
N2—C8	1.51 (5)	C16—C17	1.400 (4)
N3—C23	1.340 (3)	C16—H16A	0.9500
N3—C29	1.401 (3)	C17—C18	1.388 (4)
N3—C30	1.477 (3)	C17—C23	1.464 (4)
N4—C23	1.339 (3)	C18—H18A	0.9500
N4—C24	1.392 (3)	C19—C22	1.528 (4)
N4—H4B	0.8800	C19—C21	1.533 (4)
C1—C13	1.460 (4)	C19—C20	1.540 (4)
C2—C3	1.394 (4)	C20—H20A	0.9800
C2—C7	1.396 (4)	C20—H20B	0.9800
C3—C4	1.380 (4)	C20—H20C	0.9800
C3—H3A	0.9500	C21—H21A	0.9800
C4—C5	1.407 (4)	C21—H21B	0.9800
C4—H4A	0.9500	C21—H21C	0.9800
C5—C6	1.379 (4)	C22—H22A	0.9800
C5—H5A	0.9500	C22—H22B	0.9800
C6—C7	1.394 (4)	C22—H22C	0.9800
C6—H6A	0.9500	C24—C29	1.392 (4)
C8—C9	1.524 (16)	C24—C25	1.396 (4)
C8—H8A	0.9599	C25—C26	1.384 (4)
C8—H8B	0.9600	C25—H25A	0.9500
C9—C10	1.532 (15)	C26—C27	1.409 (4)
C9—H9A	0.9900	C26—H26A	0.9500
C9—H9B	0.9900	C27—C28	1.382 (4)
C10—C11	1.529 (15)	C27—H27A	0.9500
C10—H10A	0.9900	C28—C29	1.389 (4)
C10—H10B	0.9900	C28—H28A	0.9500
C11—C12	1.526 (15)	C30—C31	1.523 (4)
C11—H11A	0.9900	C30—H30A	0.9900
C11—H11B	0.9900	C30—H30B	0.9900
C12—H12A	0.9800	C31—C32	1.528 (4)
C12—H12B	0.9800	C31—H31A	0.9900
C12—H12C	0.9800	C31—H31B	0.9900
C8A—C9A	1.525 (8)	C32—C33	1.524 (4)
C8A—H8AA	0.9599	C32—H32A	0.9900
C8A—H8AB	0.9600	C32—H32B	0.9900
C9A—C10A	1.527 (6)	C33—C34	1.524 (5)
C9A—H9AA	0.9900	C33—H33A	0.9900
C9A—H9AB	0.9900	C33—H33B	0.9900
C10A—C11A	1.518 (6)	C34—H34A	0.9800
C10A—H10C	0.9900	C34—H34B	0.9800
C10A—H10D	0.9900	C34—H34C	0.9800
C11A—C12A	1.518 (6)		
			
Cl4—Hg—Cl3	120.68 (3)	H12D—C12A—H12E	109.5
Cl4—Hg—Cl2	108.75 (3)	C11A—C12A—H12F	109.5
Cl3—Hg—Cl2	120.54 (4)	H12D—C12A—H12F	109.5
Cl4—Hg—Cl1	102.32 (3)	H12E—C12A—H12F	109.5
Cl3—Hg—Cl1	101.25 (3)	C14—C13—C18	120.3 (2)
Cl2—Hg—Cl1	98.16 (3)	C14—C13—C1	121.9 (2)
Hg—Cl4—Cl4^i^	146.10 (4)	C18—C13—C1	117.7 (2)
C1—N1—C2	109.6 (2)	C15—C14—C13	121.4 (2)
C1—N1—H1A	125.2	C15—C14—H14A	119.3
C2—N1—H1A	125.2	C13—C14—H14A	119.3
C1—N2—C7	108.4 (2)	C16—C15—C14	117.8 (2)
C1—N2—C8A	128.6 (11)	C16—C15—C19	122.9 (2)
C7—N2—C8A	122.9 (12)	C14—C15—C19	119.2 (2)
C1—N2—C8	123 (3)	C15—C16—C17	121.2 (2)
C7—N2—C8	128 (3)	C15—C16—H16A	119.4
C23—N3—C29	108.6 (2)	C17—C16—H16A	119.4
C23—N3—C30	127.4 (2)	C18—C17—C16	120.3 (2)
C29—N3—C30	123.9 (2)	C18—C17—C23	117.5 (2)
C23—N4—C24	109.2 (2)	C16—C17—C23	122.2 (2)
C23—N4—H4B	125.4	C17—C18—C13	118.9 (2)
C24—N4—H4B	125.4	C17—C18—H18A	120.6
N1—C1—N2	109.0 (2)	C13—C18—H18A	120.6
N1—C1—C13	122.6 (2)	C22—C19—C21	108.6 (2)
N2—C1—C13	128.3 (2)	C22—C19—C15	112.1 (2)
N1—C2—C3	132.0 (2)	C21—C19—C15	108.5 (2)
N1—C2—C7	106.0 (2)	C22—C19—C20	109.1 (2)
C3—C2—C7	122.0 (2)	C21—C19—C20	109.8 (2)
C4—C3—C2	115.9 (2)	C15—C19—C20	108.7 (2)
C4—C3—H3A	122.1	C19—C20—H20A	109.5
C2—C3—H3A	122.1	C19—C20—H20B	109.5
C3—C4—C5	122.2 (3)	H20A—C20—H20B	109.5
C3—C4—H4A	118.9	C19—C20—H20C	109.5
C5—C4—H4A	118.9	H20A—C20—H20C	109.5
C6—C5—C4	121.8 (3)	H20B—C20—H20C	109.5
C6—C5—H5A	119.1	C19—C21—H21A	109.5
C4—C5—H5A	119.1	C19—C21—H21B	109.5
C5—C6—C7	116.2 (3)	H21A—C21—H21B	109.5
C5—C6—H6A	121.9	C19—C21—H21C	109.5
C7—C6—H6A	121.9	H21A—C21—H21C	109.5
N2—C7—C6	131.1 (2)	H21B—C21—H21C	109.5
N2—C7—C2	107.1 (2)	C19—C22—H22A	109.5
C6—C7—C2	121.8 (2)	C19—C22—H22B	109.5
N2—C8—C9	112 (4)	H22A—C22—H22B	109.5
N2—C8—H8A	113.5	C19—C22—H22C	109.5
C9—C8—H8A	116.9	H22A—C22—H22C	109.5
N2—C8—H8B	109.3	H22B—C22—H22C	109.5
C9—C8—H8B	96.3	N4—C23—N3	109.2 (2)
H8A—C8—H8B	107.2	N4—C23—C17	122.9 (2)
C8—C9—C10	110 (2)	N3—C23—C17	127.9 (2)
C8—C9—H9A	109.6	N4—C24—C29	106.4 (2)
C10—C9—H9A	109.6	N4—C24—C25	131.6 (2)
C8—C9—H9B	109.6	C29—C24—C25	122.0 (2)
C10—C9—H9B	109.6	C26—C25—C24	115.8 (2)
H9A—C9—H9B	108.1	C26—C25—H25A	122.1
C11—C10—C9	111.3 (18)	C24—C25—H25A	122.1
C11—C10—H10A	109.4	C25—C26—C27	122.0 (2)
C9—C10—H10A	109.4	C25—C26—H26A	119.0
C11—C10—H10B	109.4	C27—C26—H26A	119.0
C9—C10—H10B	109.4	C28—C27—C26	121.7 (2)
H10A—C10—H10B	108.0	C28—C27—H27A	119.1
C12—C11—C10	111.1 (15)	C26—C27—H27A	119.1
C12—C11—H11A	109.4	C27—C28—C29	116.3 (2)
C10—C11—H11A	109.4	C27—C28—H28A	121.8
C12—C11—H11B	109.4	C29—C28—H28A	121.8
C10—C11—H11B	109.4	C28—C29—C24	122.1 (2)
H11A—C11—H11B	108.0	C28—C29—N3	131.4 (2)
C11—C12—H12A	109.5	C24—C29—N3	106.6 (2)
C11—C12—H12B	109.5	N3—C30—C31	111.9 (2)
H12A—C12—H12B	109.5	N3—C30—H30A	109.2
C11—C12—H12C	109.5	C31—C30—H30A	109.2
H12A—C12—H12C	109.5	N3—C30—H30B	109.2
H12B—C12—H12C	109.5	C31—C30—H30B	109.2
N2—C8A—C9A	112.4 (13)	H30A—C30—H30B	107.9
N2—C8A—H8AA	107.8	C30—C31—C32	110.1 (2)
C9A—C8A—H8AA	106.1	C30—C31—H31A	109.6
N2—C8A—H8AB	108.8	C32—C31—H31A	109.6
C9A—C8A—H8AB	113.3	C30—C31—H31B	109.6
H8AA—C8A—H8AB	108.3	C32—C31—H31B	109.6
C8A—C9A—C10A	110.8 (7)	H31A—C31—H31B	108.1
C8A—C9A—H9AA	109.5	C33—C32—C31	113.9 (2)
C10A—C9A—H9AA	109.5	C33—C32—H32A	108.8
C8A—C9A—H9AB	109.5	C31—C32—H32A	108.8
C10A—C9A—H9AB	109.5	C33—C32—H32B	108.8
H9AA—C9A—H9AB	108.1	C31—C32—H32B	108.8
C11A—C10A—C9A	113.0 (6)	H32A—C32—H32B	107.7
C11A—C10A—H10C	109.0	C34—C33—C32	114.2 (2)
C9A—C10A—H10C	109.0	C34—C33—H33A	108.7
C11A—C10A—H10D	109.0	C32—C33—H33A	108.7
C9A—C10A—H10D	109.0	C34—C33—H33B	108.7
H10C—C10A—H10D	107.8	C32—C33—H33B	108.7
C12A—C11A—C10A	113.2 (5)	H33A—C33—H33B	107.6
C12A—C11A—H11C	108.9	C33—C34—H34A	109.5
C10A—C11A—H11C	108.9	C33—C34—H34B	109.5
C12A—C11A—H11D	108.9	H34A—C34—H34B	109.5
C10A—C11A—H11D	108.9	C33—C34—H34C	109.5
H11C—C11A—H11D	107.7	H34A—C34—H34C	109.5
C11A—C12A—H12D	109.5	H34B—C34—H34C	109.5
C11A—C12A—H12E	109.5		
			
C2—N1—C1—N2	−0.7 (3)	C19—C15—C16—C17	178.4 (2)
C2—N1—C1—C13	178.7 (2)	C15—C16—C17—C18	0.8 (4)
C7—N2—C1—N1	0.2 (3)	C15—C16—C17—C23	177.9 (2)
C8A—N2—C1—N1	175.5 (10)	C16—C17—C18—C13	2.0 (4)
C8—N2—C1—N1	173 (3)	C23—C17—C18—C13	−175.4 (2)
C7—N2—C1—C13	−179.2 (2)	C14—C13—C18—C17	−2.5 (4)
C8A—N2—C1—C13	−3.9 (11)	C1—C13—C18—C17	173.2 (2)
C8—N2—C1—C13	−7 (3)	C16—C15—C19—C22	−1.9 (4)
C1—N1—C2—C3	−177.2 (3)	C14—C15—C19—C22	179.3 (2)
C1—N1—C2—C7	0.9 (3)	C16—C15—C19—C21	−121.9 (3)
N1—C2—C3—C4	178.6 (3)	C14—C15—C19—C21	59.4 (3)
C7—C2—C3—C4	0.8 (4)	C16—C15—C19—C20	118.8 (3)
C2—C3—C4—C5	0.3 (4)	C14—C15—C19—C20	−60.0 (3)
C3—C4—C5—C6	−1.0 (5)	C24—N4—C23—N3	0.3 (3)
C4—C5—C6—C7	0.7 (4)	C24—N4—C23—C17	−179.1 (2)
C1—N2—C7—C6	178.9 (3)	C29—N3—C23—N4	−0.5 (3)
C8A—N2—C7—C6	3.3 (10)	C30—N3—C23—N4	−176.8 (2)
C8—N2—C7—C6	7 (3)	C29—N3—C23—C17	178.8 (2)
C1—N2—C7—C2	0.4 (3)	C30—N3—C23—C17	2.5 (4)
C8A—N2—C7—C2	−175.3 (9)	C18—C17—C23—N4	37.0 (3)
C8—N2—C7—C2	−172 (2)	C16—C17—C23—N4	−140.3 (3)
C5—C6—C7—N2	−178.0 (3)	C18—C17—C23—N3	−142.3 (3)
C5—C6—C7—C2	0.4 (4)	C16—C17—C23—N3	40.4 (4)
N1—C2—C7—N2	−0.8 (3)	C23—N4—C24—C29	0.1 (3)
C3—C2—C7—N2	177.6 (2)	C23—N4—C24—C25	179.3 (3)
N1—C2—C7—C6	−179.5 (2)	N4—C24—C25—C26	−179.7 (3)
C3—C2—C7—C6	−1.2 (4)	C29—C24—C25—C26	−0.6 (4)
C1—N2—C8—C9	115 (5)	C24—C25—C26—C27	0.9 (4)
C7—N2—C8—C9	−74 (6)	C25—C26—C27—C28	−0.2 (4)
N2—C8—C9—C10	175 (4)	C26—C27—C28—C29	−0.8 (4)
C8—C9—C10—C11	177 (4)	C27—C28—C29—C24	1.1 (4)
C9—C10—C11—C12	−178 (3)	C27—C28—C29—N3	−179.8 (2)
C1—N2—C8A—C9A	102.1 (16)	N4—C24—C29—C28	178.9 (2)
C7—N2—C8A—C9A	−83 (2)	C25—C24—C29—C28	−0.4 (4)
N2—C8A—C9A—C10A	−175.0 (15)	N4—C24—C29—N3	−0.4 (3)
C8A—C9A—C10A—C11A	179.7 (15)	C25—C24—C29—N3	−179.7 (2)
C9A—C10A—C11A—C12A	−178.1 (9)	C23—N3—C29—C28	−178.6 (3)
N1—C1—C13—C14	136.1 (3)	C30—N3—C29—C28	−2.2 (4)
N2—C1—C13—C14	−44.6 (4)	C23—N3—C29—C24	0.6 (3)
N1—C1—C13—C18	−39.6 (4)	C30—N3—C29—C24	177.0 (2)
N2—C1—C13—C18	139.8 (3)	C23—N3—C30—C31	−105.7 (3)
C18—C13—C14—C15	0.3 (4)	C29—N3—C30—C31	78.5 (3)
C1—C13—C14—C15	−175.2 (2)	N3—C30—C31—C32	175.7 (2)
C13—C14—C15—C16	2.3 (4)	C30—C31—C32—C33	173.0 (2)
C13—C14—C15—C19	−178.8 (2)	C31—C32—C33—C34	−65.8 (3)
C14—C15—C16—C17	−2.9 (4)		

Symmetry code: (i) −*x*+2, −*y*, −*z*+2.

### Hydrogen-bond geometry (Å, º)

**Table:**

*D*—H···*A*	*D*—H	H···*A*	*D*···*A*	*D*—H···*A*
N1—H1*A*···Cl1	0.88	2.30	3.171 (2)	171
N4—H4*B*···Cl1	0.88	2.35	3.224 (2)	170
C3—H3*A*···Cl3	0.95	2.90	3.803 (3)	160
C6—H6*A*···Cl2^ii^	0.95	2.56	3.492 (3)	169
C18—H18*A*···Cl1	0.95	2.85	3.331 (4)	113
C25—H25*A*···Cl4^i^	0.95	2.96	3.664 (3)	132
C28—H28*A*···Cl4^iii^	0.95	2.91	3.796 (3)	156
C30—H30*A*···Cl4^iii^	0.99	2.77	3.627 (3)	145

Symmetry codes: (i) −*x*+2, −*y*, −*z*+2; (ii) −*x*+1, −*y*, −*z*+1; (iii) −*x*+1, −*y*, −*z*+2.
